# Transcriptome and Metabolome Profiles Reveal the Underlying Mechanism of Fat Deposition Changes in Three-Way Crossbred Yak for High-Quality Beef Production

**DOI:** 10.3390/ani15172599

**Published:** 2025-09-04

**Authors:** Xiukai Cao, Wenxiu Ru, Jie Cheng, Le Sun, Nan Zhang, Lawang Zhaxi, Renzeng Dunzhu, Fengbo Sun, Kai Yang, Yue’e Gao, Xixia Huang, Bizhi Huang, Hong Chen

**Affiliations:** 1 College of Animal Science, Xinjiang Agricultural University, Urumqi 830052, China; cxkai0909@163.com (X.C.);; 2 Joint International Research Laboratory of Agriculture and Agri-Product Safety of Ministry of Education of China, Yangzhou University, Yangzhou 225009, China; 3 Key Laboratory of Animal Genetics, Breeding and Reproduction of Shaanxi Province, College of Animal Science and Technology, Northwest A&F University, Yangling 712100, China; 4 Jiangsu Key Laboratory of Sericultural and Animal Biotechnology, Jiangsu University of Science and Technology, Zhenjiang 212100, China; 5 Tibet Autonomous Region Center for Animal Disease Control and Prevention, Lhasa 850032, China; 6 Yunnan Academy of Grassland and Animal Science, Kunming 650212, China

**Keywords:** transcriptomics, metabolomics, LC-MS/MS, fat deposition, yak

## Abstract

Yajiangxue cattle (XF) is three-way crossbred cattle developed specifically for producing high-quality beef in the Tibetan Plateau by introducing the bloods of Tibetan yellow cattle (HF) and Angus cattle into Tibetan yak (MF). In the present study, we mainly focused on fat deposition and metabolism changes and used RNA-seq and LC-MS/MS-based metabolomics to partially explain the meat quality improvement in Yajiangxue cattle. Differential expression analysis revealed 1762, 2949, and 2931 different expression genes in XF vs. HF, XF vs. MF, and XF vs. cattle–yak (PF), respectively, such as *BMP2*, *WISP2*, *FGF1*, *IL1B*, *IL6*, and *WNT5B*. Immune response, oxidation–reduction processes, and fatty acid metabolism were markedly enriched. Furthermore, an initial identification revealed 319 metabolites using positive ion mode and 289 metabolites using negative ion mode in bovine adipose tissue across four breeds/populations. Of these, 143 were differential metabolites in positive ion mode, while 166 were in negative ion mode. The main pathways of metabolism affected by breed/population were unsaturated fatty acid biosynthesis, tryptophan and tyrosine biosynthesis, primary bile acid biosynthesis, cholesterol metabolism, beta-alanine metabolism, etc. Similarly, both the transcriptome and the metabolome results highlighted fatty acid metabolism. These results could help elucidate the biological mechanisms involved in fat deposition and identify valuable biomarkers for specific metabolite accumulation.

## Simple Summary

Yaks are important animals that live in the high mountains of Tibet, providing food and income for local people. However, yak meat quality could be improved to meet growing consumer demands. We created a new type of cattle called Yajiangxue by carefully breeding yaks with two other cattle breeds over multiple generations. This breeding approach combined the best traits from each animal: the ability to survive in harsh mountain conditions from yaks, fewer calving difficulties from Tibetan yellow cattle, and better meat quality from Angus cattle. We studied the fat tissue from these animals using advanced laboratory techniques to understand what makes their meat better. We found that Yajiangxue cattle had higher levels of healthy fats and beneficial compounds compared to their parent breeds. These cattle also showed better resistance to cellular damage and had more favorable fat composition that could improve meat flavor and nutritional value. The fat tissue contained more of certain good fatty acids that are beneficial for human health, as well as compounds that act as natural antioxidants. Our research helps explain why this new crossbred cattle produces higher quality meat and provides scientific evidence for developing better livestock breeding programs. This work could help farmers in Tibet and similar regions produce more nutritious and flavorful beef while maintaining animals well-adapted to challenging mountain environments.

## 1. Introduction

The Tibetan Plateau, a vast, elevated region often referred to as the ‘Roof of the World’, is the highest and most expansive plateau globally. Its average altitude of 5 km and area of roughly 3 million square kilometers have earned it the nickname the ‘Third Pole’ [1,2]. Yaks (Bos grunniens), a sacred religious symbol, evolved special physiological features for the harsh climatic environments of the Tibetan Plateau characterized by extreme cold, low oxygen levels, and intense ultraviolet radiation. It was essential for sustaining life on the plateau, providing resources like transportation, food, shelter, and fuel and intricately linked to the Tibetan nationality. Nowadays, yak husbandry is thriving and has greatly improved Tibetans’ socio-cultural life.

Due to the rising demand for beef, approximately 14 million domestic yaks in China have become a valuable alternative beef source, providing a significant boost to the Tibetan economy. Improving yak meat production and quality is one of major goals of the yak industry for the next few decades, given customers’ growing concerns about the health and nutritional benefits of their food [3]. Despite the success of pure-breeding in increasing meat production, improving meat quality traits is challenging due to their low-to-moderate heritability (0.14–0.33), including fat composition and fat content [4]. Crossbreeding accompanied with heterosis is an efficient strategy for generating desirable improvements in both of the two traits [5]. Heterosis means the performance of crossbred descendants is better than the expected average of their parents for a particular trait. Studies have shown that the specific breeds used in crossbreeding systems significantly impact the quality of yak meat [6]. To address the limitations of two-way crossbreeding systems, such as calving difficulties caused by large size disparities between breeds like Simmental or Holstein and yaks, which often lead to dystocia and increased mortality of dams and calves [7], we developed a novel three-way crossbreeding system to produce Yajiangxue cattle (XF). By using Tibetan yellow cattle (HF) as an intermediate parental breed, this system reduces dystocia risks due to HF’s medium-sized stature, which facilitates easier calving compared to direct Angus–yak crosses. Furthermore, Angus was used as a terminal sire to enhance heterosis and integrate superior meat quality and rapid growth by breeding with half-yak (F1) females called cattle–yak (PF) from ♂Tibetan yellow cattle × ♀Tibetan yak (MF). Yajiangxue cattle exhibited rapid growth, high productivity, excellent meat quality, and exceptional adaptability to the plateau environment, giving them a comprehensive advantage over MF, HF, PF, and Angus cattle. Here, we mainly focused on fat deposition changes and used omics analyses of transcription and metabolization to partially explain the meat quality improvement in XF.

It is widely believed that fat deposition (composition and content) is a crucial indicator of meat quality. Meatier cattle were those that were small and blocky, representing selection for fat and not muscle. Japanese Wagyu has good quality, obvious marbling, and strong fat deposition capacity [8]. Fatty acids, the metabolites of fat, are important factors affecting the flavor and nutrition of beef [9]. Fatty acids are composed of monounsaturated fatty acids (MUFAs), saturated fatty acids (SFAs), polyunsaturated fatty acids (PUFAs), nutraceutical fatty acids (n-3, n-6 PUFAs), and functional unsaturated fatty acids (FUFAs). Yak kidney fat is rich in functional fatty acids and has a valuable composition. Specifically, fifteen fatty acids were identified in yak kidney fat, comprising 49.68% SFAs and 48.58% UFAs. This composition indicates the potential for developing yak fat into commercial products for the food industry [10]. A comparison of lipid profiles revealed that plateau PF and MF muscle contains higher levels of phospholipids enriched with long-chain polyunsaturated fatty acids than plain cattle muscle, suggesting superior lipid nutritional quality [11]. The lipid composition of yak shanks and flanks may also differ significantly, with the metabolic pathways of sphingolipids and glycerophospholipids playing key roles in driving these differences in lipid components [12]. The differences in fatty acid composition in yak meat under grazing and stall feeding regimes, as well as the regulatory mechanisms of the feeding system on fatty acid content in yak meat, have been explored [13,14]. Therefore, analyzing meat quality traits by comparing fat tissue across different cattle breeds holds great significance.

However, most of existing studies have focused on production traits, and the systematic comparison of fat deposition among yak and its crossbred descendants of different generations has yet to be conducted. Integrative omics analysis of transcription and metabolization has been demonstrated as an efficient method for uncovering the functions of unknown genes and establish gene–metabolite relationships, which outperform individual omics and the candidate gene method [15]. Transcriptome analysis estimates the gene mRNA expression profile through RNA-Seq. Metabolomics is a comprehensive approach that involves both the quantitative and qualitative analysis of low-molecular-weight metabolites. These metabolites, which are the final products of cellular processes, have the potential to directly affect the phenotype. A range of advanced metabolomic technologies is employed for the determination and analysis of small molecules, including 1H nuclear magnetic resonance (1H NMR), liquid chromatography–mass spectrometry (LC-MS), and gas chromatography–mass spectrometry (GC-MS) [16,17,18,19,20]. The combination of transcriptomics and non-targeted metabolomics has unveiled the mechanisms underlying follicular atresia in Chinese buffalo [21]. Using a similar method, another integrated analysis provided insights into the intricate molecular regulatory network underlying meat quality in Enshi black pigs [22]. Additionally, a metabolomic and transcriptomic study was conducted to investigate the alterations in metabolic and immune responses of steers exposed to heat stress [23]. Here, we integrated RNA-Seq and LC-MS/MS of fat tissues from yak and its crossbred descendants of different generations to find potential valuable biomarkers of beef quality, which may facilitate further molecular breeding of yak.

## 2. Materials and Methods

### 2.1. Ethics Statement

The protocols and animals used in this study were approved by the Faculty Animal Policy and Welfare Committee of Northwest A&F University, NWAFAC1008.

### 2.2. Animals and Data Collection

MF (Niangya yak breed) were crossbred with HF to produce PF hybrids (1/2 yak). Subsequently, Angus cattle were used as terminal sires to artificially inseminate PF, resulting in the creation of XF cattle (1/4 yak). During the summer and autumn, these animals were grazed on alpine pastures in Tibet. In spring and winter, they were housed in barns with ad libitum access to feed and water, following the NY/T 815-2004 standard [24]. The diet was formulated with a concentrate-to-roughage ratio of 35:65. Roughages included alfalfa hay (50%) and wheat straw (15%), while concentrates consisted of corn flour (14%), wheat bran (6.83%), soybean meal (4.9%), cottonseed meal (3.85%), fermented distiller’s grains (3.5%), NaCl (0.17%), and a premix (1.75%). The diet provided a net energy for maintenance and fattening of 7.04 MJ/kg, with 11.42% crude protein, 0.68% total calcium, 0.39% total phosphorus, 27.96% neutral detergent fiber, and 15.02% acid detergent fiber, ensuring adequate nutrition for growth, fat deposition, and adaptation to the Tibetan Plateau’s high-altitude environment. Animals were randomly selected at 25 months of age, housed in pens, and serially slaughtered at 36 months of age for experimental purposes and subsequent meat quality analysis. A total of 23 fasted animals were randomly selected for quality analysis, including MF (n = 6, 3 males and 3 females), HF (n = 5, 2 males and 3 females), PF (n = 6, all females), and XF (n = 6, 3 males and 3 females). The animals were fasted for 24 h prior to slaughter, and the carcasses were subsequently chilled for 24 h at 4 °C. After slaughter, data on head weight, front hoof weight, back hoof weight, tare weight, liver weight, heart weight, lung weight, spleen weight, kidney weight, stomach weight, visceral fat, oxtail, bullwhip, small intestine, large intestine, intestinal fat, marbling score, and fat color were collected for analysis. Subcutaneous fat samples were collected from the backfat over the longissimus dorsi muscle (between the 12th and 13th ribs), a standard site for assessing fat deposition in beef cattle. These samples were collected immediately after slaughter, within 10 min, and were then rapidly snap-frozen in liquid nitrogen to preserve RNA integrity. Both the marbling score and fat color were evaluated according to the Chinese national standard GB/T 29392-2022 [25]. Marbling score was assessed using a 5-point scale, where 1 indicates scarce marbling and 5 indicates abundant marbling. Fat color was evaluated using an 8-point scale, where 1 represents white and 8 represents yellow.

### 2.3. RNA Extraction and Transcriptome Analysis

Total RNA was extracted from subcutaneous fat samples using Trizol reagent (Invitrogen, Carlsbad, CA, USA) according to the manufacturer’s guidelines. RNA integrity was assessed using an Agilent 2100 Bioanalyzer (Agilent Technologies, Santa Clara, CA, USA) and agarose gel electrophoresis, while RNA purity was measured with a NanoPhotometer spectrophotometer (Thermo Scientific, Waltham, MA, USA). Trimmomatic v0.32 was used to remove sequence adapters and low-quality reads, followed by FastQC v0.10.1 quality assessment. The sequencing reads were then aligned to the ARS-UCD1.2 cattle reference genome using HISAT2 v2.2.4. Differential gene expression analysis was conducted with DESeq2 v1.24.0, and genes with a false discovery rate (FDR) value less than 0.05 were considered differentially expressed genes (DEGs). To perform gene set enrichment analysis (GSEA), all the genes were ranked based on fold-change and significance derived from the differential analysis [26]. GSEA was conducted using mSigDB C2 gene sets and curated gene sets.

### 2.4. Non-Targeted LC-MS/MS Metabolomics Analysis

The pretreatment of LC-MS/MS samples was carried out according to a previous report [27]. LC-MS/MS analysis (Novogene Co., Ltd., Beijing, China) was performed using a UHPLC system equipped with a Hypesil Gold column (2.1 mm × 100 mm, 1.9 µm) coupled to a Q Exactive HF-X mass spectrometer. The mobile phase consisted of water containing 0.1% formic acid (for positive ion mode) and 5 mmol/L ammonium acetate (for negative ion mode) (A) and methanol (B), with a flow rate of 0.2 mL/min. The elution gradient was 2% B for 1.5 min; 2% to 100% B over 12.0 min; 100% B for 14.0 min; 100% to 2% B over 14.1 min; and 2% B for 17 min. The Exactive HF-X mass spectrometer was operated in both positive and negative polarity modes with parameters set as 10 arb (auxiliary gas), 40 arb (sheath gas), 3.2 kV (spray voltage), and 320 °C (capillary temperature).

Compound Discoverer 3.1 (Thermo Fisher Scientific, Waltham, MA, USA) was used for the alignment and picking of peak and metabolite quantitation with raw data to obtain qualitative and quantitative results of metabolites. Based on additive ions, fragment ions, and molecular ion peaks, the total spectral intensity obtained by normalizing the peak intensities was employed to calculate the molecular formula. Peaks were subsequently compared with the MassList, mzVault, and mzCloud databases to achieve precise qualitative and relative quantitative results. Following identification, metabolites were functionally annotated using the online databases HMDB, KEGG, and Lipid Maps. Multivariate statistical analyses, including principal component analysis (PCA) and partial least squares discriminant analysis (PLS-DA), were performed with MetaX software (version 1.0.4) to evaluate the processed data [28]. With R2 and Q2 values used to assess the goodness of fit, we validated PLS-DA models through permutation tests. To investigate the changes in metabolite composition of bovine fat caused by different breeds/populations, various comparisons between XF and HF, XF and MF, XF and PF were conducted, respectively. Metabolites were classified as differential if they had a variable importance in projection (VIP) > 1, a *t*-test *p*-value < 0.05, and a fold change (FC) ≥ 1.2 or FC ≤ 0.833. The relative metabolite levels were determined using Z-score plots, and the trends in metabolite changes across the four breeds/populations were analyzed. The KEGG database was adopted to predict the functions of these metabolites.

### 2.5. Integrated Analysis of the Transcriptome and Metabolome

To assess the relationship between DEGs and differential metabolites (DMs), we calculated pairwise Pearson correlation coefficients using R (Version 4.3.2). All identified DEGs and DMs were mapped to the KEGG pathway database (https://www.genome.jp/kegg/pathway.html, accessed on 20 June 2022) to find the shared pathways information using Microsoft Excel, and the main biochemical pathways and signal transduction pathways in which the DEGs and DMs were determined.

### 2.6. Statistical Analyses

One-way ANOVA was used to compare the performances of economic traits of yak and its crossbred descendants of different generations. After conducting the one-way ANOVA, we used least significant difference (LSD) post hoc test for multiple comparisons among the four groups. * *p* < 0.05, ** *p* < 0.01, *** *p* < 0.001.

## 3. Results

### 3.1. XF Possess Enhanced Growth Characteristics and Meat Quality

**Figure 1:**
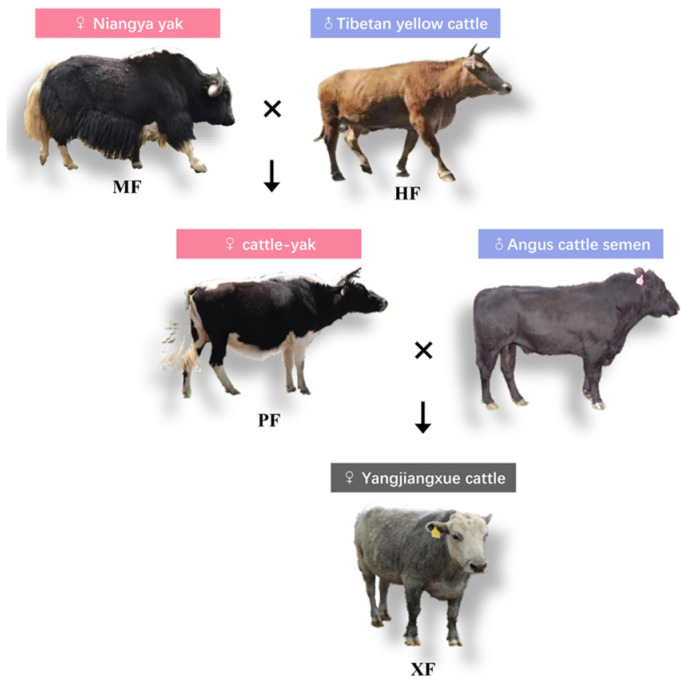
Three-way cross system for producing Yajiangxue cattle with high meat production and quality.

We analyzed the growth traits and meat quality of Niangya yak and its crossbred descendants of different generations (Figure 1). Notably, a direct comparison with Angus cattle was not feasible due to the use of artificial insemination with Angus semen, which prevented Angus samples under identical environmental and management conditions from being obtained. The breeds significantly affected head weight, front hoof weight, back hoof weight, tare weight, liver weight, heart weight, lung weight, spleen weight, kidney weight, stomach weight, visceral fat, oxtail, bullwhip, small intestine, large intestine, and intestinal fat (*p* < 0.01). These growth traits were much higher in XF than in HF, MF, and PF (*p* < 0.01) (Table 1). Additionally, we compared the marbling score and fat color among the four breeds, and the marbling score of XF was found to be higher than other breeds on average (Table 1). These data suggest that XF developed through a three-way cross system on the Tibetan Plateau have high yield, rapid growth, and superior meat quality.

### 3.2. Identification of Differentially Expressed Genes (DEGs)

**Figure 2:**
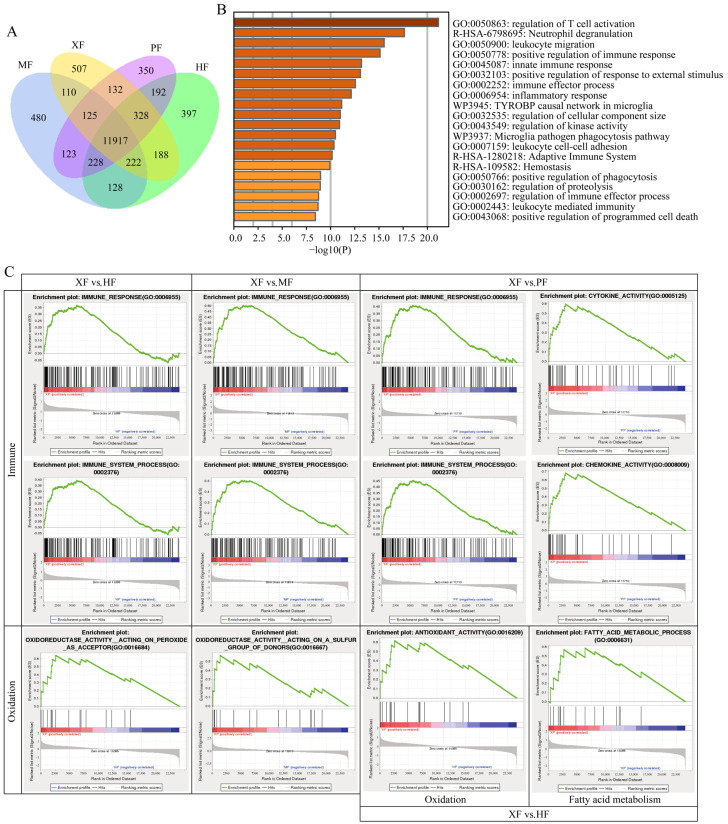
Gene annotation analyses. (**A**) Venn diagram of expressed genes between all breed samples. (**B**) GO and KEGG of 360 XF-specific highly expressed genes by Metascape. (**C**) GSEA analysis.

RNA-Seq was performed to assess the subcutaneous fat of the four breeds. The raw reads per sample ranged from 38,050,738 to 48,497,648, with an average of 45,116,929 reads. The average clean reads per sample was 44,341,967. The average Q20 and Q30 percentages were 96.88% and 91.85%, respectively, indicating high sequencing quality. The average unique mapping rate was 82.76%, indicating a high quality of alignment. Detailed information is given in Table S1. The expression abundance of all genes is listed in Table S2. We found 1762, 2949, and 2931 DEGs in XF vs. HF, XF vs. MF, and XF vs. PF, respectively, as shown in volcano plots (Figure S1). More specifically, there were 937 upregulated and 825 downregulated genes in XF vs. HF, 1594 upregulated and 1355 downregulated genes in XF vs. MF, and 1587 upregulated genes and 1344 downregulated genes in XF vs. PF. These DEGs had clearly different expression patterns, as visualized in the heatmap shown in Figure S1. Among these DEGs, there were 507 exclusively expressed genes in XF, 397 genes in HF, 480 genes in MF, and 350 genes in PF (Figure 2A). The lipid-related DEGs in XF are significantly higher than in the HF, MF, and PF groups, such as *HADH*, and XF is significantly higher than HF and PF for genes such as *ESR1* and *APOL3*, which can promote growth and fat deposition.

### 3.3. DEGs Are Enriched in Immune, Oxidation, and Fat Metabolism

First, gene ontology (GO) analysis was performed for functional annotation of DEGs, including molecular function (MF), cellular component (CC), and biological process (BP). For the XF vs. HF group (*p* < 0.05; Table S3), the DEGs were grouped into 55 significantly different subcategories, encompassing 21 MF terms, 6 CC terms, and 28 BP terms. For the MF category, 2.5% of the genes (20 of 788 genes) were located in enzyme binding (GO:0005488). For the CC category, 11.6% of the genes (31 of 268 genes) were annotated to the extracellular region (GO:0044421). For the BP category, 4.5% (22/489), 4.3% (21/489 genes), and 8.6% (42/489) of the genes were involved in the immune response (GO:0006955), immune system process (GO:0002376), and oxidation–reduction process (GO:0055114), respectively.

For the XF vs. MF group (*p* < 0.05; Table S4), the DEGs were grouped into 37 significantly different subcategories, including 14 MF terms, 5 CC terms, and 18 BP terms. For the MF category, 6.5% of the genes (86 of 1320 genes) were located in oxidoreductase activity (GO:0016491). For the CC category, 11.16% of the genes (52 of 466 genes) were annotated into the extracellular region (GO:0005576). For the BP category, 3.9% (32/823) and 3.8% (31/823) of the genes were involved in the immune system process (GO:0002376) and immune response (GO:0006955), respectively. For the XF vs. PF group (*p* < 0.05; Table S5), the DEGs were grouped into 79 significantly different subcategories, including 33 MF terms, 21 CC terms, and 25 BP terms. For the MF category, 4.2% (55/1310) of the genes were located in the structural constituent of the ribosome (GO:0003735). For the CC category, 21.4% (98/459) of the genes were annotated into the mitochondrion (GO:0005739). For the BP category, 7.7% (63/813) and 7.9% (64/813) of the genes were involved in the peptide biosynthetic process (GO:0043043) and peptide metabolic process (GO:0006518), respectively.

To further explore the potential biological significance of phenotype divergence, the identified DEGs were mapped to various pathways using KEGG bioinformatics databases. For XF vs. HF group (*p* < 0.01; Table S6), the enrichment analysis revealed that the top enriched terms were primarily related to immune-related pathways, such as phagosome, Th1/Th2/Th17 cell differentiation, NF-kappa B signaling pathway, human T-cell leukemia virus 1 infection, and hematopoietic cell lineage, as well as fat-related metabolism, such as type I diabetes mellitus and the thyroid hormone signaling pathway. For XF vs. MF group (*p* < 0.01; Table S7), enrichment analysis revealed that the top enriched terms were primarily related to immune-related pathways, such as phagosome, hematopoietic cell lineage, NF-kappa B signaling pathway, leukocyte transendothelial migration, and B cell receptor signaling pathway, as well as fat-related metabolism, such as the PPAR signaling pathway. For XF vs. PF group (*p* < 0.01; Table S8), enrichment analysis revealed that the top enriched terms were primarily related to immune-related pathways, such as phagosome and Th1/Th2/Th17 cell differentiation, as well as fat-related metabolism, such as FA metabolism, UFA biosynthesis, FA elongation, FA degradation, and non-alcoholic fatty liver disease. Interestingly, 360 genes in XF were expressed at significantly higher levels than in HF, MF, and PF. These genes, including *BMP2*, *WISP2*, *FGF1*, *IL1B*, *IL6*, *WNT5B*, *TNFSF14*, *FST*, *CCBE1*, and *HSPB1*, were mainly involved in immune-related pathways, such as regulation of T cell activation, neutrophil degranulation, and leukocyte migration (*p* < 0.01; Table S9, Figure 2B).

GSEA suggested that, compared with HF, XF showed upregulated genes enriched in oxidoreductase activity acting on peroxide as an acceptor (GO:0016684), antioxidant activity (GO:0016209), immune system process (GO:0002376), immune response (GO:0006955), and FA metabolic process (GO:0006631). Compared with MF, XF showed upregulated genes enriched in oxidoreductase activity acting on a sulfur group of donors (GO:0016667), purine nucleoside binding (GO:0001883), purine ribonucleoside binding (GO:0032550), immune system process (GO:0002376), and immune response (GO:0006955). Compared with PF, XF showed upregulated genes enriched in cytokine activity (GO:0005125), chemokine activity (GO:0008009), G protein coupled receptor binding (GO:0001664), immune response (GO:0006955), and immune system process (GO:0002376) (*p* < 0.05; Figure 2C). Overall, the immune response, oxidation–reduction, and fatty acid metabolism were markedly enriched in XF compared with HF, MF, and PF, showing that XF have good meat quality, resistance to oxidation, and strong plateau adaptability.

### 3.4. Identification of Differential Metabolites

**Figure 3:**
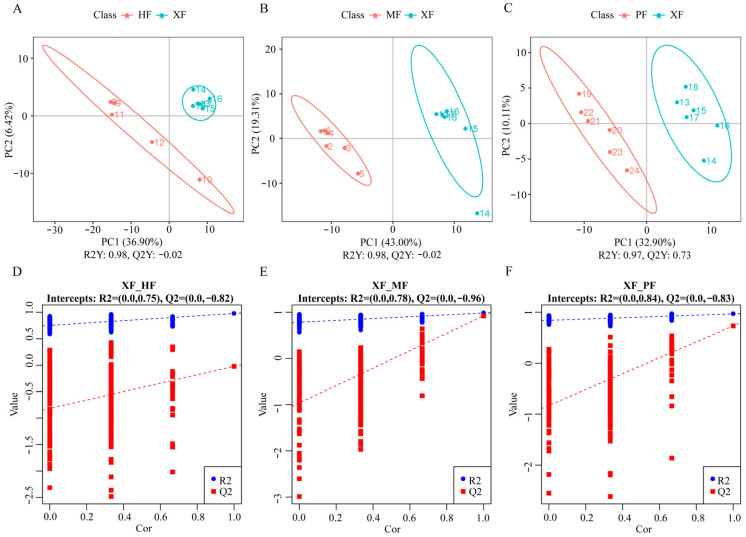
The PLS-DA (**A**–**C**) and its models validated by permutation tests (**D**–**F**) between different breed groups in negative ion mode. R2Y = 0.98, Q2Y = −0.02 in XF vs. HF; R2Y = 0.98, Q2Y = 0.92 in XF vs. MF; R2Y = 0.97, Q2Y = 0.73 in XF vs. PF.

To ensure the stability and quality of our metabolomics data, we used three quality control samples, which were analyzed as biological replicates. The stability of the entire detection process was assessed by calculating the Pearson correlation coefficient between the relative quantitative values of the metabolites in the quality control samples. Highly repeatable and reliable data were produced, given the observation that the Pearson correlation coefficient among quality control samples ranged from 0.991 to 0.995. Subsequently, PLS-DA was employed as a supervised pattern recognition method for multivariate statistical analysis. The negative ion mode clearly differentiates between any two breeds/populations (Figure 3), and the positive ion mode is presented in Figure S2. For the cross-validation, Q2Y indicates the predictive ability of models, while R2Y indicates the interpretation ability of the models. The R2Y values exceeded the Q2Y values, indicating that the models exhibited good cumulative interpretation and predictive abilities. To prevent model overfitting, subsequent permutation tests were conducted (Figure 3 and Figure S2). The R2 values (blue line) consistently exceeded the Q2 values (red line), suggesting a low risk of overfitting in these models.

**Figure 4:**
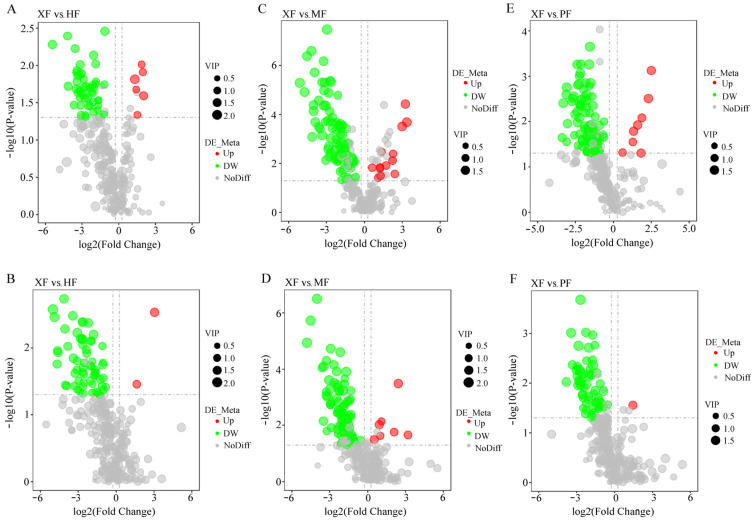
Analysis of metabolome profile diversity between different breeds. (**A**) XF vs. HF in neg model; (**B**) XF vs. HF in pos model; (**C**) XF vs. MF in neg model; (**D**) XF vs. MF in pos model; (**E**) XF vs. PF in neg model; (**F**) XF vs. PF in neg model. Each dot corresponds to a metabolite, with red dots indicating significantly upregulated metabolites and green dots indicating significantly downregulated metabolites. Gray dots represent metabolites with no significant differential expression. The size of each dot reflects the VIP numeric value.

A total of 319 and 289 metabolites were initially identified in positive and negative ion modes, respectively. After filtering out metabolites with no significant changes, 143 and 166 DMs remained in positive and negative ion modes, respectively. Results showed that 8, 20, and 9 metabolites were significantly upregulated and 109, 175, and 145 metabolites were downregulated in the comparison of XF vs. HF, XF vs. MF, XF vs. PF, respectively (Figure 4, Table S10).

Lipid-related metabolites play an essential role in meat quality. In XF vs. HF (Table S11), two differential fatty acids and conjugate metabolites including 16-hydroxyhexadecanoic acid (HMDB0006294) and trans-10-heptadecenoic acid (HMDB0244268) experienced an up-trend. Both positive and negative ions also detected oxidized glutathione (HMDB0003337,). Vitamin B2 (HMDB0000244), taurine (HMDB0000251), and DL-lanthionine (HMDB0251521) were also detected. In XF vs. MF (Table S11), two differential fatty acids and conjugate metabolites including 16-hydroxyhexadecanoic acid (HMDB0006294) and palmitic acid (HMDB0000220) were detected. Seven organic acids and derivative metabolites including taurine (HMDB0000251), oxidized glutathione (HMDB0003337), Asp-Glu (HMDB0028752), cyclohexaneacetic acid (HMDB0031403), O-phosphorylethanolamine (HMDB0000224), N-acetyl-L-leucine (HMDB0011756), and DL-lanthionine (HMDB0251521pos) were identified. Two glycerophospholipids, LPC 20:3 (LMGP01050139) and PE (18:0/18:1) (LMGP02010301), were also detected. In XF vs. PF (Table S11), the differential fatty acid and conjugate metabolite is trans-10-heptadecenoic acid (HMDB0244268), and the differential pregnane steroid metabolite is pregnenolone (HMDB0000253,). Two differential glycerophosphocholines, LPC 20:3 (LMGP01050139) and PC (18:0/18:1) (LMGP01010761), were identified. 13(S)-HOTrE (cpd:C16316, map00592, alpha-Linolenic acid metabolism) was detected in alpha-linolenic acid metabolism.

**Figure 5:**
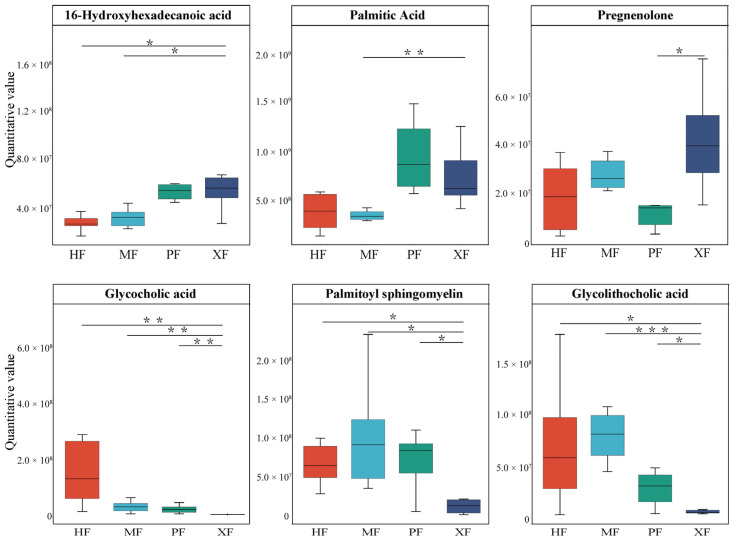
The relative peak intensities of pivotal metabolites affecting meat flavor and quality across four breeds/populations. * *p* < 0.05, ** *p* < 0.01, *** *p* < 0.001.

Notably, several metabolites of fatty acid and umami amino acids influencing beef quality and flavor were significantly altered in XF (Figure 5). 16-hydroxyhexadecanoic acid in XF was more significantly increased than in HF and MF, trans-10-heptadecenoic acid in XF was more significantly increased than in HF and PF, and palmitic acid in XF was more significantly increased than in MF. The differential steroid and steroid derivative metabolite pregnenolone experienced an upward trend in the breed comparison of XF vs. PF, while glycolithocholic acid, glycocholic acid, and palmitoyl sphingomyelin decreased in XF. The above findings suggest that XF have good meat quality and are rich in nutrition and flavor.

### 3.5. DMs Are Enriched in Fatty Acid Biosynthesis and Hormone Metabolism

The top pathways of differential metabolites of XF vs. HF, XF vs. MF, and XF vs. PF are outlined in Figure S3. DMs identified in the XF and HF were most significantly enriched in the oxytocin signaling pathway and caffeine metabolism (Figure S3A,B). UFA biosynthesis was the most significant pathway detected in the breed/population comparison of XF and MF (Figure S3C,D). Antifolate resistance, the AMPK signaling pathway, the longevity regulating pathway, pyrimidine metabolism, and the sphingolipid signaling pathway were significant pathways detected in the comparison of XF and PF (Figure S3E,F). These findings support the roles of FAs and hormones in yak growth and meat quality.

### 3.6. Integrated Analysis Reveals Promising Candidates Associated with Meat Quality

**Figure 6:**
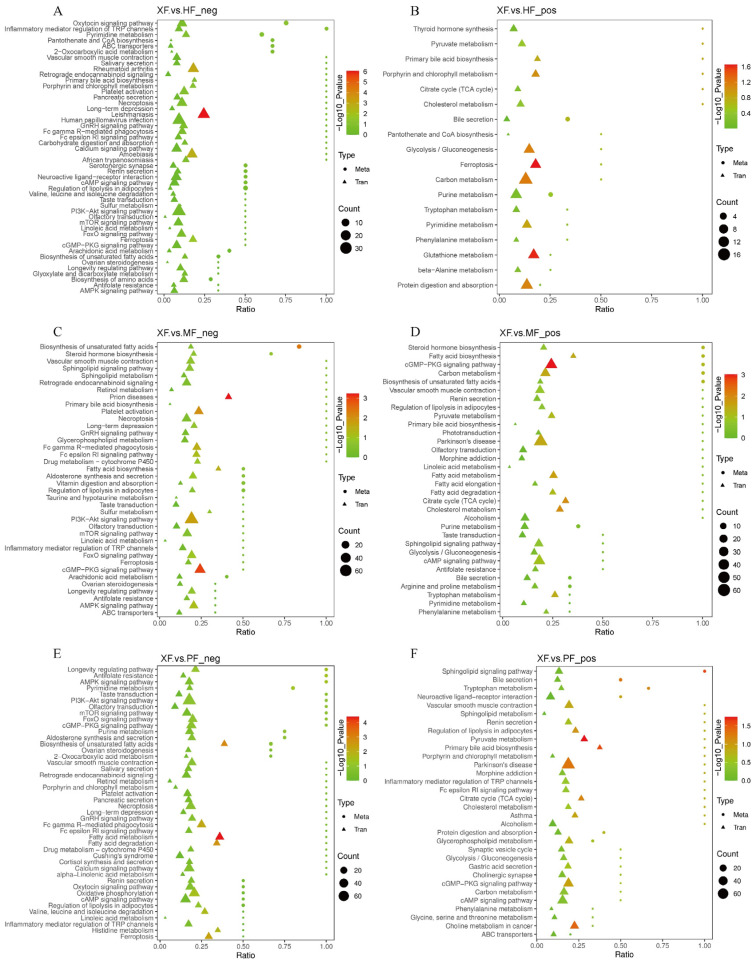
Integrated KEGG analysis of metabolome and transcriptome profiles across different breed groups. (**A**) XF vs. HF_neg; (**B**) XF vs. HF_pos; (**C**) XF vs. MF_neg; (**D**) XF vs. MF_pos; (**E**) XF vs. PF_neg; (**F**) XF vs. PF_pos.

**Figure 7:**
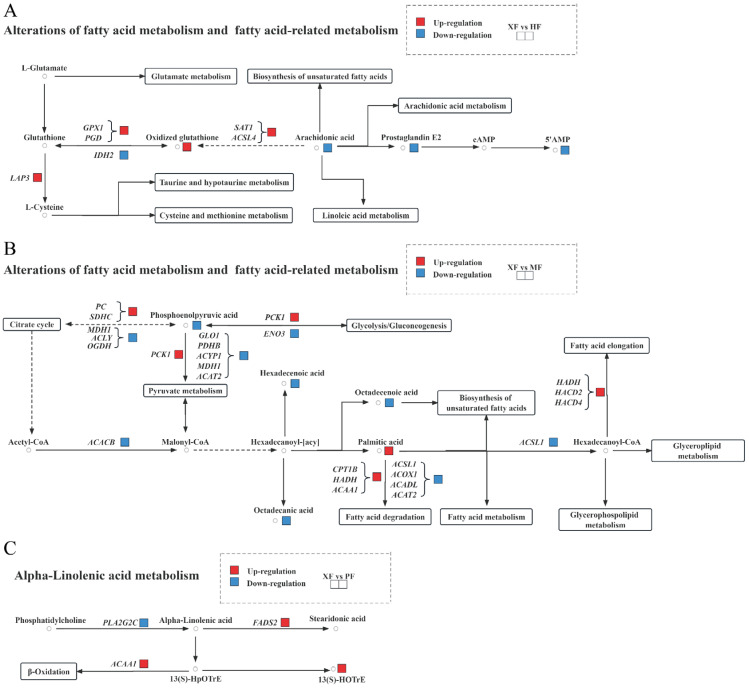
Alterations in fatty acid metabolism in different breed groups. (**A**) Schematic representation of the genes and metabolites involved in fatty acid metabolism (XF vs. HF). (**B**) Schematic representation of the genes and metabolites involved in fatty acid metabolism (XF vs. MF). (**C**) Schematic representation of the genes and metabolites involved in alpha-linolenic acid metabolism (XF vs. PF). Blue indicates significant downregulation. Red indicates significant upregulation. Genes are italicized to differentiate them from metabolites.

To elucidate the correlation between DEGs and DMs, we conducted an integrated analysis of metabolome and transcriptome profiles. The complete results are presented in Tables S11–S17, and the top integration KEGG pathways are visualized in Figure 6. We extracted integrated KEGG pathways associated with meat quality or flavor, and the significant correlations are summarized in Table 2. In detail, in the comparison between XF and HF (*p* < 0.05; Figure 7A, Tables S12 and S13), four differential metabolites—oxidized glutathione, arachidonic acid, prostaglandin E2, and 5’AMP—involved in FA metabolism, were significantly associated with several key genes, including *LAP3*, *GPX1*, *PGD*, *SAT1*, *ACSL4*, and *IDH2*. Interestingly, these metabolites and genes are all involved in glutamate metabolism, biosynthesis of UFAs, arachidonic acid metabolism, taurine and hypotaurine metabolism, cysteine and methionine metabolism, and linoleic acid metabolism, indicating their critical role in fat formation during the breeding process.

In the comparison between XF and MF (*p* < 0.05; Figure 7B, Tables S14 and S15), five differential metabolites responsible for fatty acid metabolism—phosphoenolpyruvic acid, hexadecenoic acid, octadecanic acid, octadecenoic acid, and palmitic acid—were significantly correlated with several key genes, including *PC*, *SDHC*, *MDH1*, *ACLY*, *OGDH*, *PCK1*, *ENO3*, *GLO1*, *PDHB*, *ACYP1*, *ACAT2*, *ACACB*, *CPT1B*, *HADH*, *ACAA1*, *ACSL1*, *ACOX1*, *ACADL*, *HADH*, *HACD2*, and *HACD4*. Interestingly, these metabolites and genes are all involved in fatty acid metabolism, indicating their critical role in fat formation during the breeding process. Meanwhile, these results imply that citrate cycle, glycolysis/gluconeogenesis, pyruvate metabolism, FA degradation, FA metabolism, biosynthesis of UFAs, FA elongation, glycerophospolipid metabolism, and glyceroplipid metabolism might affect fat formation in XF compared with MF, XF, and PF.

In the comparison between XM and PM (*p* < 0.05; Figure 7C, Tables S16 and S17), one differential metabolite responsible for alpha-linolenic acid metabolism, 13(S)-HOTrE, was significantly correlated with several key genes, including *PLA2G2C*, *FADS2*, and *ACAA1*. This differential metabolite was responsible for β-oxidation. These results imply that alpha-linolenic acid metabolism might affect fat formation in XF compared with PF.

## 4. Discussion

As efficient utilizers of highland herbage resources, yaks are vital to the Tibetan Plateau’s livestock industry. The yak industry has flourished in the past decade, significantly boosting the regional economy. However, ensuring consumer satisfaction by evaluating the quality of yaks and their hybrids is a pressing concern. Given the relatively low heritability of fat deposition, introducing exogenous blood with favorable traits may be a promising approach from a genetic and breeding perspective.

In this study, a three-way cross system was created with Angus cattle, a globally renowned beef breed known for its exceptional marbled appearance. By introducing HF as the parental F0, this three-way cross system aims to mitigate the calving difficulties associated with two-way crossbreeding between Angus and yak. The native breed, with a medium-sized stature compared to Angus and yak, provides an advantage for the crossbred offspring in handling the challenging foraging conditions of the Qinghai–Tibetan Plateau [29]. All in all, the excellent reproductive performance of Angus and HF makes them well-suited for yak hybridization on the Tibetan Plateau. This is the first three-way cross system designed for high-quality beef production on the Tibetan Plateau. Our results demonstrate that this system effectively combines optimal fat deposition with alpine adaptability, rapid growth, high yield, and excellent meat quality. Our results showed that XF individuals generally grow faster and reach larger sizes than yaks, particularly in later stages of development. The introduction of Angus into the breeding program prevented inbreeding depression or heterosis loss commonly observed in backcrosses to yaks or local cattle breeds. As a result, XF populations exhibited enhanced growth performance from birth compared to PF individuals.

Among these desirable traits, meat quality is crucial for economic success in the beef cattle industry. Numerous factors influence meat quality, including tenderness, hardness, color, flavor, muscle fiber characteristics, oxidative stability, and fat composition and content [4]. Meat quality is a complex trait influenced by various physicochemical characteristics, including tenderness, pH, color, FAs composition, intramuscular fat content, and sensory attributes [30]. It is widely believed that fat deposition (composition and content) is a crucial indicator of meat quality. Adipose-derived secretory factors, such as lipokines and adipokines, play a role in regulating skeletal muscle development and maintaining homeostasis and may have potential in human health and husbandry production [31]. Therefore, analyzing fat tissue from different cattle breeds is crucial for understanding and improving meat quality traits.

Extensive research has demonstrated that animal fat deposition is a complex biological process influenced by various transcriptional factors, including SREBP-1c, STAT5, PPARγ, KLF family, and C/EBP family, which have been shown to play significant roles in promoting adipogenesis [32]. Differences in gene expression related to fat deposition have been extensively characterized using RNA-Seq. In this research, lipid-related DEGs such as *HADH* were found to be significantly higher in XF than in HF, MF, and PF, with *ESR1* and *APOL3* being significantly higher in XF than in HF and PF, which can promote growth and fat deposition. HADH protein affects lipid deposition in pigs, as revealed through iTRAQ-based proteomic analysis [33]. *ESR1*, related to fat deposition, was significant higher in fat tail than small tail tissues [34]. *APOL3*, which plays a role in lipid transport and metabolism, was identified as a highly duplicate gene in the beef breeds. It was found to have a higher copy number in Angus compared to Holstein, Hereford, and Nelore [35]. The *APOL3* gene is also suggested to be a candidate gene for IMF deposition [36]. Notably, key genes such as *BMP2* and *FGF1* were significantly upregulated in XF compared to HF, MF, and PF. *BMP2* is known to promote adipogenesis by inducing preadipocyte differentiation through the activation of the PPARγ signaling pathway, as evidenced by studies showing reduced fat accumulation in *BMP2* knockout models [37]. Similarly, *FGF1* enhances lipid accumulation and insulin sensitivity in adipose tissue, with overexpression experiments demonstrating increased fat deposition in transgenic mice [38]. These findings suggest that the upregulation of *BMP2* and *FGF1* in XF contributes to enhanced fat deposition, supporting the observed improvements in marbling score and meat quality.

A total of 360 genes were found to be significantly higher in XF than in HF, MF, and PF. Enrichment analysis indicated that the top enriched terms were primarily associated with immune-related pathways, showing that XF have strong immune capacity and environmental suitability. The enrichment of immune pathways in XF, such as NF-kappa B signaling and cytokine activity, suggests a link to fat deposition, as inflammation can modulate adipogenesis. For instance, the inflammatory factor IL6, upregulated in XF, has a controversial role in adipose tissue. It may promote chronic inflammation, leading to impaired adipogenesis and insulin resistance in obesity [39], yet it also potentiates BMP2-induced adipogenesis by activating pathways like p38 MAPK, enhancing lipid accumulation in mesenchymal stem cells [40]. This dual role implies that IL6 contributes to the observed fat deposition balancing immune responses and adipogenic processes, supporting improved meat quality traits like marbling. In the XF vs. HF and XF vs. MF groups, oxidoreductase activity (GO:0016491) and oxidation–reduction (GO:0055114) were enriched, showing that the oxidoreductase response plays an important role in the change in F0 and F2. In the XF vs. HF group comparison, XF were found to have higher antioxidant activity compared to HF. For example, for *GPX1* and *CAT*, their expression levels in the two populations were both XF > HF. *GPX1* belongs to the glutathione peroxidase family, which reduces organic hydroperoxides and hydrogen peroxide (H_2_O_2_) by glutathione, thereby protecting cells from oxidative damage. Similarly, *CAT* encodes catalase, a crucial antioxidant enzyme involved in the body’s defense against oxidative stress [41]. As well as the immune response and oxidation–reduction being markedly enriched, fatty acid metabolism was also important. Fatty acid metabolism and purine-related metabolism were associated with umami flavor [18]. Compared with HF and MF, the beef umami in XF has a better taste. Collectively, these findings provided a novel molecular understanding of the observed improvement in XF from the perspective of environmental suitability, oxidation–reduction, and good fat deposition.

The metabolic profile of chicken meat was analyzed through 1H NMR spectroscopy [17,18]. A comparative metabolomics analysis of milk components was conducted using LC-MS/MS technology, focusing on Chinese Holstein cows versus Italian Mediterranean buffaloes [19]. A combined LC-MS and GC-MS approach was employed to analyze the metabolite characteristics of thigh meat from fast- and slow-growing broilers at market age [20]. Since LC-MS/MS detected more metabolites than GC-MS/MS, the effects of breed need to be explored across a wider metabolite spectrum. Here, non-targeted LC-MS/MS technology was used to determine the metabolite composition of bovine fat in XF, HF, MF, and PF.

The composition of dietary FAs is also closely linked to human health. Trans-10-heptadecenoic acid, 16-hydroxyhexadecanoic acid, and palmitic acid are fatty acids and conjugates, among which trans-10-heptadecenoic acid is an unsaturated fatty acid and essential fatty acid, which cannot be synthesized by the body and therefore must be obtained through the diet [42]. 16-hydroxyhexadecanoic acid (also known as 16-hydroxypalmitic acid, ω-hydroxypalmitic acid, lanopalmitic acid, or juniperic acid; C_16_H_32_O_3_), a hydroxylated derivative of palmitic acid, was significantly increased in XF compared to HF and MF. While our study highlights the elevated levels of this metabolite in XF, we acknowledge that no direct association with meat quality phenotypes, such as marbling score, has been established in the current literature or our data. However, hydroxy fatty acids, including 16-hydroxyhexadecanoic acid, are known to influence fat deposition [43], contribute to the formation of flavor compounds like lactones in meat [44], and improve insulin sensitivity [45], suggesting potential indirect contributions to meat quality and nutritional value in XF. Bovine fat is an important source of unsaturated fatty acid. Dietary supplementation of food with 16-hydroxyhexadecanoic acid, trans-10-heptadecenoic acid, and palmitic acid has been used as a potential method to supplement saturated and monounsaturated FAs in foods. Intramuscular fat refers to the lipids stored within the muscle, primarily consisting of cholesterol, phospholipids, and triglycerides [46,47]. Lipid oxidation significantly contributes to the development of meat flavor, with early studies indicating that phospholipids are more likely to generate flavor compared to triglycerides [48]. Three differential glycerophospholipid metabolites—PE (18:0/18:1), LPC (20:3), and PC (18:0/18:1)—experienced a significant uptrend in the breed comparison of XF vs. MF, XF vs. MF, and XF vs. PF, respectively, showing that XF has better lipid deposition compared to MF and PF, inheriting from HF or Angus.

Regarding umami amino acids, Asp-Glu was more significantly increased in XF than in MF [49]. Amino acids not only serve as important nutritional components for humans but also act as precursors to the flavor of cooked meat and contribute to its umami taste. In total, 37 different amino acids, peptides, and analogues were detected in bovine fat through metabonomic. DL-lanthionine was more significantly increased in XF than in HF and MF. The biological value of fat largely depends on the presence of digestible proteins that contain essential amino acids, which the human body cannot synthesize. In the comparison of amino acid pathways between XF and HF, MF, and PF, the identified differential metabolites were enriched in pathways related to isoleucine, leucine, and valine biosynthesis; tryptophan, tyrosine, and phenylalanine biosynthesis; and tryptophan, phenylalanine, and beta-alanine metabolism. Among these, isoleucine, leucine, valine, tryptophan, tyrosine, and phenylalanine are essential amino acids in the related pathways. We recognize that umami amino acids are more directly associated with meat flavor when evaluated in muscle tissue rather than subcutaneous fat. However, our analysis of umami amino acids such as Asp-Glu in subcutaneous fat provides valuable insights into the systemic metabolic differences among breeds/populations. Subcutaneous adipose tissue serves as an active metabolic organ that influences overall carcass composition and indirectly affects meat quality through several mechanisms. Adipose tissue acts as a reservoir for amino acids and their metabolites, which can be mobilized and transported to muscle tissue during meat aging and processing [50]. The metabolic activity of subcutaneous fat influences the overall amino acid profile in circulation, potentially affecting muscle metabolism [51]. Subcutaneous fat metabolites can serve as biomarkers reflecting the breed’s overall metabolic capacity for flavor compound production. While our subcutaneous fat results cannot directly predict muscle umami intensity, they indicate the breed’s inherent metabolic potential for amino acid synthesis and metabolism, suggesting that XF may have superior capacity for developing desirable flavor compounds. Future studies should indeed analyze muscle tissue directly to validate these metabolic differences in terms of actual eating quality and sensory attributes.

Metabolic pathway analysis is a valuable approach for examining the direct internal relationships between metabolites, allowing the reconstruction of biochemical reaction networks [52]. KEGG pathway analysis found that biosynthesis of UFAs; biosynthesis of phenylalanine, tryptophan, and tyrosine; metabolism of tryptophan; biosynthesis of primary bile acid; citrate cycle (TCA cycle); and metabolism of cholesterol, beta-alanine, linoleic acid, alpha-linoleic acid, and purine were the significant pathways of bovine fat metabolism affected by breed/population. Alanine metabolism and purine metabolism have been identified as key pathways influencing the meat flavor of Wuding chicken, suggesting that breed differences may account for the variation in metabolic pathways [18].

Integrating multi-omics data can aid in revealing potential gene functions related to specific metabolite accumulation. Using RNA-seq data, we performed the analysis of Pearson correlation between metabolite profiles and gene expression during the cattle breeding process. In this study, we paid attention to the differential metabolites enriched in the most significant pathways across the four breeds/populations and investigated the corresponding key genes in each breed. These genes were considered as the most promising potential markers for explaining the influence of breed on the metabolic profiles of cattle. In the XF vs. HF and XF vs. MF groups, oxidoreductase activity (GO:0016491) and the oxidation–reduction process (GO:0055114) were enriched, showing that oxidoreductase response played an important role in the change in F0 and F2. Raw meat is prone to oxidative deterioration during processing and storage due to chemical reactions. Nevertheless, the inclusion of antioxidants can mitigate the rate and extent of this oxidative deterioration, thereby prolonging the shelf life of meat products. Meat is a source of various endogenous antioxidants and bioactive compounds, such as creatine, carnosine, carnitine, glutathione, taurine, and ubiquinone [53]. High levels of taurine, oxidized glutathione, and vitamin B2 were detected in the XF group, and these compounds are known to have antioxidant properties. Taurine is recognized for its significant potential in the development of functional foods. It helps prevent liver steatosis through the inhibition of lipogenesis and promotion of energy expenditure, and it also mitigates oxidative damage by reducing ROS levels and stabilizing the mitochondrial membrane [54]. Glutathione reductase (GR) reduces oxidized glutathione (GSSG) to glutathione (GSH) by utilizing NADPH, as previously described [55]. The oxidized form of glutathione, GSSG, is known for its antioxidant properties and detoxification capabilities, and has potential therapeutic applications for numerous diseases, including cancer and chronic diseases [56]. In a previous study, it was observed that an antioxidant-rich diet led to a decrease (*p* < 0.0001) in GSH and GSH/GSSG values, and an increase (*p* < 0.0001) in GSSG levels in the liver of animals compared to those not receiving such a diet. Furthermore, the activity of the glutathione peroxidase enzyme was found to be higher (*p* < 0.01) in animals on an antioxidant-rich diet [57]. These findings suggest that the modulation of glutathione levels through dietary interventions may have significant implications for the prevention and treatment of various diseases. The antioxidant nature of vitamin B2 can help safeguard the body from oxidative stress, particularly by mitigating lipid peroxidation and oxidative damage resulting from reperfusion [58]. Our study observed increased taurine, oxidized glutathione, and vitamin B2 levels in the fat of XF than that in HF and MF, suggesting that different breeds may have distinct metabolic mechanisms. These findings demonstrated that the XF group exhibited optimal oxidation resistance, suggesting that this breed/population may contribute to improved meat quality and extended shelf life.

Taurine, oxidized glutathione, and vitamin B2 also have reducibility and were higher in XF than in HF and MF. Taurine is a particularly noteworthy amino acid found in fat. It is a crucial component of meat quality, contributing to taste and also playing essential physiological roles in humans [59]. Studies in animal models have shown that taurine supplementation can help alleviate metabolic disorders such as diabetes, hypertension, hyperlipidemia, and obesity [60]. Oxidized glutathione was detected in both positive and negative ion mode and contributes to certain flavor characteristics, especially in meat [61]. It is commonly assumed that vitamin B2 intake is overestimated based on dietary intakes, as compared to the levels measured in biochemical status indexes. In fact, studies have found that B2 intakes are often more than 5% below the recommended intake, with young women and seniors being particularly affected. To address this issue, increasing the B2 content in beef would not only help meet recommended intake levels but would also provide labeling advantages [62]. Moreover, research has suggested that B2 may indirectly impact cancer development through its influence on folate, highlighting the potential importance of increasing B2 content in beef [63]. In conclusion, XF cattle meat could be introduced to the market.

## 5. Conclusions

In summary, XF is a three-way crossbreed with rapid growth, high yield, superior meat quality, and robust adaptability to plateau conditions, giving them a comprehensive advantage over HF, MF, PF, and Angus cattle. In the present study, we mainly focused on fat deposition and fat metabolism changes and used transcriptome sequencing and LC-MS/MS-based metabolomics to partially explain the meat quality improvement in XF. Overall, differential expression analysis revealed 1762, 2949, and 2931 DEGs in XF vs. HF, XF vs. MF, and XF vs. PF, respectively. Lipid-related DEGs such as *HADH* are significantly higher in XF than in HF, MF, and PF, while *ESR1* and *APOL3* are significantly higher in XF than in HF and PF, which can promote growing and fat deposition. The DEGs were markedly enriched in immune response, oxidation–reduction, and fatty acid metabolism, including *BMP2*, *WISP2*, *FGF1*, *IL1B*, *IL6*, and *WNT5B*. Additionally, 319 metabolites were initially identified in bovine adipose tissue using positive ion mode and 289 metabolites using negative ion mode, including 143 differential metabolites in positive ion mode and 166 differential metabolites in negative ion mode across four breeds/populations. Overall, fat in XF has the highest level of trans-10-heptadecenoic acid, indicating a lower risk of cardiovascular disease. Asp-Glu, which contributes to fat flavor, presented an upward trend in XF. Vitamin B2, taurine, and oxidized glutathione, which are involved in antioxidant processes, presented an upward trend in XF. The main pathways of XF fat metabolism influenced by breed were UFA biosynthesis; phenylalanine, tyrosine, and tryptophan biosynthesis; tryptophan metabolism; primary bile acid biosynthesis; citrate cycle (TCA cycle); cholesterol metabolism; beta-alanine metabolism; linoleic acid metabolism; alpha-linoleic acid metabolism; and purine metabolism. Integrated analysis of the RNA-Seq and metabolome data uncovered potentially functional genes that impact chemical compositions and metabolic pathways. These findings provide insights into the biological processes driving fat deposition and help identify important biomarkers of specific metabolite accumulation. Additionally, future studies should prioritize the quantification of the differential metabolites identified in this research to accurately reflect the nutritional composition of bovine fat. It is also essential to investigate potential gene markers and their roles in the accumulation of specific metabolites at the cellular level. In summation, XF fat offers higher energy, a stronger umami flavor, and optimal oxidation resistance. Similarly, the transcriptome results and the metabolome results also focus on fatty acid metabolism. Integrated analysis of the metabolome and transcriptome can provide valuable insights into the biological mechanisms underlying fat deposition and facilitate the identification of biomarkers associated with specific metabolite accumulation.

## Figures and Tables

**Table 1:** Growth traits and meat quality of yak and its crossbred descendants.

Economic Traits	Breeds/Populations (LSM ± SE)				*p*
	MF (n = 6)	HF (n = 5)	XF (n = 6)	PF (n = 6)	
Head weight	27.91 ± 1.10 ^Bc^	19.74 ± 1.32 ^Cd^	44.44 ± 1.23 ^Aa^	32.51 ± 2.13 ^Bb^	0.000
Front hoof weight	4.53 ± 0.26 ^Bb^	2.76 ± 0.31 ^Cc^	8.07 ± 0.29 ^Aa^	5.24 ± 0.50 ^Bb^	0.000
Back hoof weight	4.53 ± 0.28 ^Bb^	2.82 ± 0.34 ^Cc^	8.06 ± 0.32 ^Aa^	5.00 ± 0.55 ^BCb^	0.000
Tare weight	31.46 ± 1.85 ^Bc^	20.22 ± 2.22 ^Cd^	56.27 ± 2.06 ^Aa^	38.84 ± 3.58 ^Bb^	0.000
Heart weight	1.74 ± 0.13 ^Bb^	1.06 ± 0.15 ^Cc^	4.93 ± 0.14 ^Aa^	2.27 ± 0.25 ^Bb^	0.000
Liver weight	5.74 ± 0.50 ^Bb^	3.92 ± 0.60 ^Bc^	11.57 ± 0.56 ^Aa^	7.22 ± 0.97 ^Bb^	0.000
Lung weight	4.89 ± 0.48 ^Bb^	2.45 ± 0.58 ^Cc^	7.53 ± 0.54 ^Aa^	5.13 ± 0.94 ^ABCb^	0.000
Spleen weight	0.58 ± 0.08 ^Bc^	0.36 ± 0.10 ^Bc^	1.69 ± 0.09 ^Aa^	1.20 ± 0.16 ^Ab^	0.000
Kidney weight	0.86 ± 0.07 ^Bb^	0.61 ± 0.09 ^Bc^	1.52 ± 0.08 ^Aa^	1.01 ± 0.14 ^ABbc^	0.000
Stomach weight	15.34 ± 1.14 ^Bb^	12.37 ± 1.37 ^Bb^	32.00 ± 1.27 ^Aa^	17.39 ± 2.21 ^Bb^	0.000
Visceral fat	na	na	53.94 ± 4.27 ^Aa^	11.82 ± 4.57 ^Bb^	0.001
Oxtail	0.85 ± 0.43 ^Bb^	0.69 ± 0.52 ^Bb^	4.31 ± 0.48 ^Aa^	1.46 ± 0.84 ^ABb^	0.000
Bullwhip	0.99 ± 2.03 ^b^	1.82 ± 2.32 ^b^	10.67 ± 1.64 ^a^	na	0.027
Small intestine	5.68 ± 0.57 ^Bb^	3.59 ± 0.68 ^Bc^	8.27 ± 0.64 ^Aa^	5.39 ± 1.10 ^ABabc^	0.000
Large intestine	6.61 ± 1.66 ^Bb^	4.61 ± 1.99 ^Bb^	15.96 ± 1.85 ^Aa^	5.60 ± 3.21 ^ABb^	0.000
Intestinal fat	3.76 ± 1.97 ^Bb^	3.14 ± 2.37 ^Bb^	27.42 ± 2.20 ^Aa^	13.34 ± 3.82 ^ABb^	0.000
Marbling score	0.00 ± 0.00 ^Bb^	1.00 ± 0.00 ^Bb^	3.17 ± 0.60 ^Aa^	1.78 ± 0.31 ^Aa^	0.000
Fat color	7.00 ± 0.00 ^Aa^	6.00 ± 0.00 ^Bb^	2.00 ± 0.00 ^Cc^	6.33 ± 0.21 ^Bb^	0.000

Note: Different letters in the same row indicate significant differences among groups (uppercase letters for *p* < 0.01, lowercase letters for *p* < 0.05). LSM, least square means; SE, standard error; MF, yak; HF, Tibetan yellow cattle; XF, Yajiangxue cattle; PF, cattle–yak; na, not available.

**Table 2:** Significant correlations between metabolite profiles and gene expressions potentially associated with meat quality.

Metabolites	Groups	Trend	KEGG Pathway	Significantly Related Genes (*p* < 0.05)
oxidized glutathione	XF vs. HF	up	ferroptosis	*CP* (gene id 514194), *FTL* (286861), *FTH1* (281173), *ACSL4* (536628), *CYBB* (281112), *LOC788801* (788801), *TF* (280705), *novel.1303*, *ATG* (5532686), *SAT1* (508861)
oxidized glutathione	XF vs. HF	up	glutathione metabolism	*RRM1* (505537), *GPX1* (281209), *LAP3* (781648), *GSTA1* (777644), *PGD* (514939), *CHAC1* (505991), *IDH2* (327669), *LOC100295687* (100295687), *RRM2B* (528960), *ODC1* (281365), and *GSTA3* (768055)
palmitic acid	XF vs. MF	up	fatty acid biosynthesis	*HSD17B8* (532422), *ACACB* (515338), *CBR4* (533020), *RPP14* (515208), *ACSF3* (509209), and *ACSL1* (537161)
palmitic acid	XF vs. MF	up	fatty acid metabolism	*FADS2* (521822), *ACOX1* (513996), *HACD4* (618814), *ACAT2* (512044), *HSD17B8* (532422), *ACADL* (614508), *CBR4* (533020), *HADH* (532785), *CPT1B* (509459), *RPP14* (515208), *HACD2* (613886), *LOC613570* (613570), *ACSF3* (509209), *ELOVL1* (540348), *ACSL1* (537161), and *ACAA1* (508324)
sedoheptulose 1,7-bisphosphate	XF vs. MF	up	carbon metabolism	*LOC101902656* (101902656), *HK3* (510616), *PSPH* (533630), *PDHB* (613610), *ACOX1* (513996), *ENO3* (540303), *ACAT2* (512044), *PRPS2* (537688), *HIBCH* (535883), *MDH2* (281306), *HK2* (788926), *PSAT1* (533044), *ALDH6A1* (327692), *IDH3B* (613338), *MDH1* (535182), *LOC788293* (788293), *PCCA* (614302), *GPI* (280808), *GOT2* (286886), *GLYCTK* (507949), *OGDH* (534599), *LOC614208* (614208), *FBP1* (513483), *PHGDH* (505103), *IDH3G* (614145), *SDHC* (327696), *LOC616200* (616200), *GCSH* (317723), and *PC* (338471)
13(S)-HOTrE	XF vs. PF	up	alpha-linolenic acid metabolism	*ACAA1* (508324), *PLA2G2C* (504978), and *FADS2* (521822)

## Data Availability

The raw data supporting the conclusions of this article will be made available by the authors on request.

## Supplementary Materials

The following supporting information can be downloaded at: https://www.mdpi.com/article/10.3390/ani15172599/s1, Figure S1: Preliminary analysis of transcriptome profiles of XF, HF, MF and PF. Figure S2: The PLS-DA and its models validated by permutation tests between different breed groups in positive ion mode. Figure S3: The KEGG pathways of differential metabolites between the four breed groups. Table S1: RNA-Seq_statistic. Table S2: RNA-Seq_expression. Table S3: GO analysis for functional annotation of DEGs of XF vs. HF. Table S4: GO analysis for functional annotation of DEGs of XF vs. MF. Table S5: GO analysis for functional annotation of DEGs of XF vs. PF. Table S6: KEGG analysis for DEGs of XF vs. HF. Table S7: KEGG analysis for DEGs of XF vs. MF. Table S8: KEGG analysis for DEGs of XF vs. PF. Table S9: GO annotation for XF specific genes. Table S10: Statistic of different metabolites. Table S11: Different lipid metabolites. Table S12: Integrated KEGG analysis of XF vs. HF in negative ions. Table S13: Integrated KEGG analysis of XF vs. HF in positive ions. Table S14: Integrated KEGG analysis of XF vs. MF in negative ions. Table S15: Integrated KEGG analysis of XF vs. MF in positive ions. Table S16: Integrated KEGG analysis of XF vs. PF in negative ions. Table S17: Integrated KEGG analysis of XF vs. PF in positive ions.

## Abbreviations

The following abbreviations are used in this manuscript:

DEG	differentially expressed gene
DM	differential metabolite
FC	fold change
FUFA	functional unsaturated fatty acid
GC-MS	gas chromatography–mass spectrometer
GO	gene ontology
GSEA	gene set enrichment analysis
HF	Tibetan yellow cattle
LC-MS	liquid chromatography–mass spectrometry
LC-MS/MS	liquid chromatography with tandem mass spectrometry
MF	Tibetan yak
MUFA	monounsaturated fatty acid
PCA	principal component analysis
PF	cattle–yak
PLS-DA	partial least squares discriminant analysis
PUFA	polyunsaturated fatty acid
SFA	saturated fatty acid
VIP	variable importance in projection
XF	Yajiangxue cattle
