# Supplementary material for: Transcriptome and Metabolome Profiles Reveal the Underlying Mechanism of Fat Deposition Changes in Three-Way Crossbred Yak for High-Quality Beef Production
Source: Animals (Basel). 2025 Sep 4;15(17):2599. doi: 10.3390/ani15172599 (PMC12427193; doi:10.3390/ani15172599)
Supplement: Supplementary file 1 [file animals-15-02599-s001.zip › Supplementary Figures.pdf]

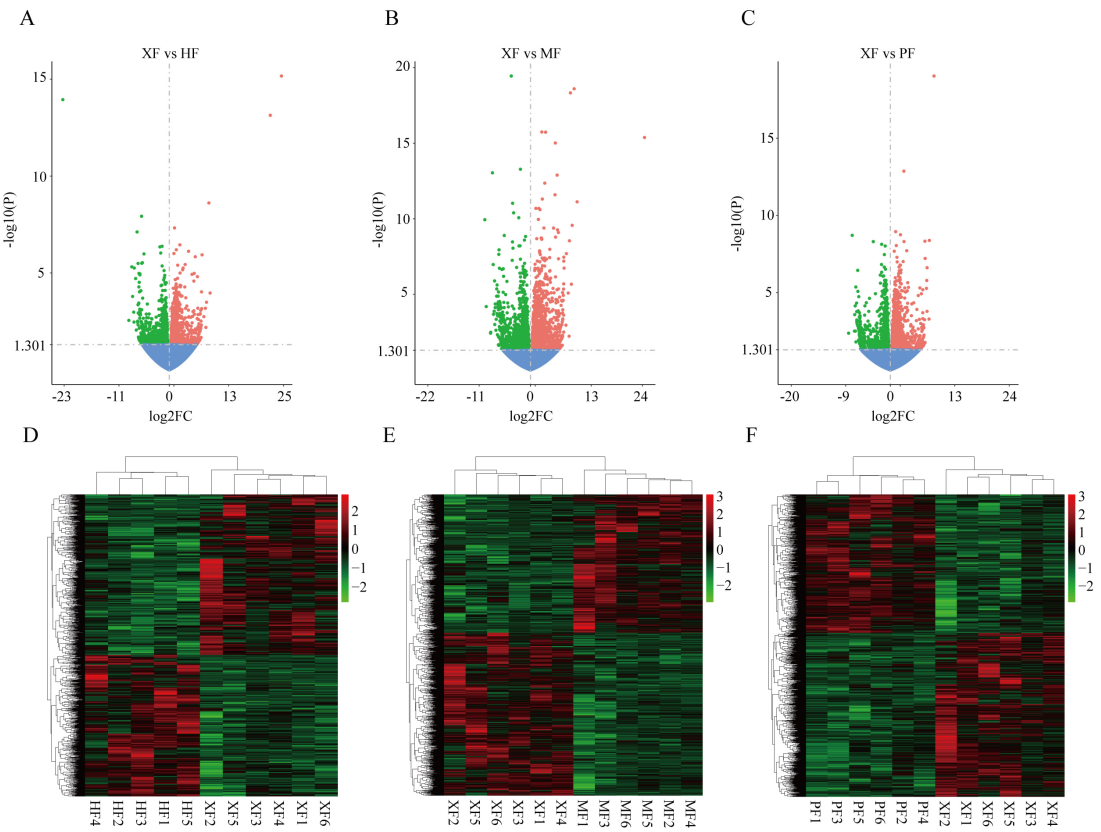

Figure S1. Preliminary analysis of transcriptome profiles of XF, HF, MF and PF. (**A**) Volcano plots of DEGs in XF vs HF. (**B**) Volcano plots of DEGs in XF vs MF. (**C**) Volcano plots of DEGs in XF vs PF. The green dots represent the downregulated expressed genes; the red dots represent the upregulated expressed genes; the blue dots represent the non-differentially expressed genes. (**D**) Heat map of DEGs in XF vs MF. (**E**) Heat map of DEGs in XF vs PF. (**F**) Heat map of DEGs in XF vs PF.

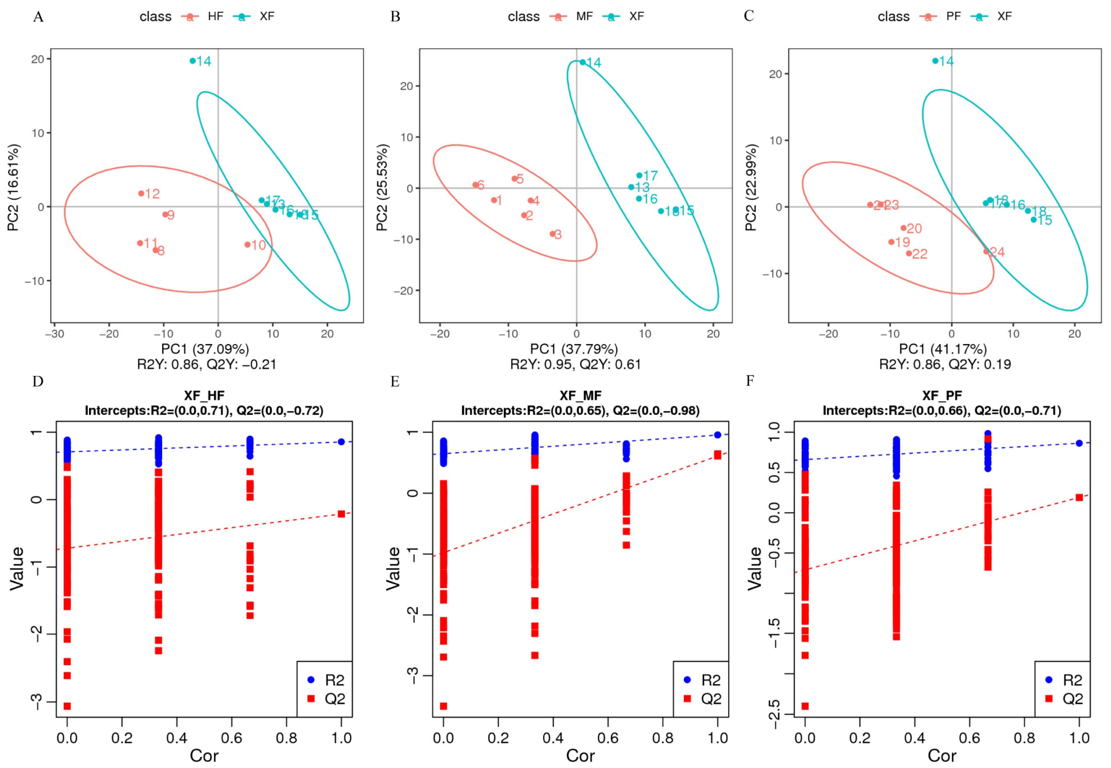

Figure S2. The PLS-DA (A, B, C) and its models validated by permutation tests (D, E, F) between different breed groups in positive ion mode. R2Y = 0.86, Q2Y = -0.21 in XF vs HF; R2Y = 0.95, Q2Y = 0.61 in XF vs MF; R2Y = 0.86, Q2Y = 0.19 in XF vs PF. PLS-DA models validated by permutation tests.

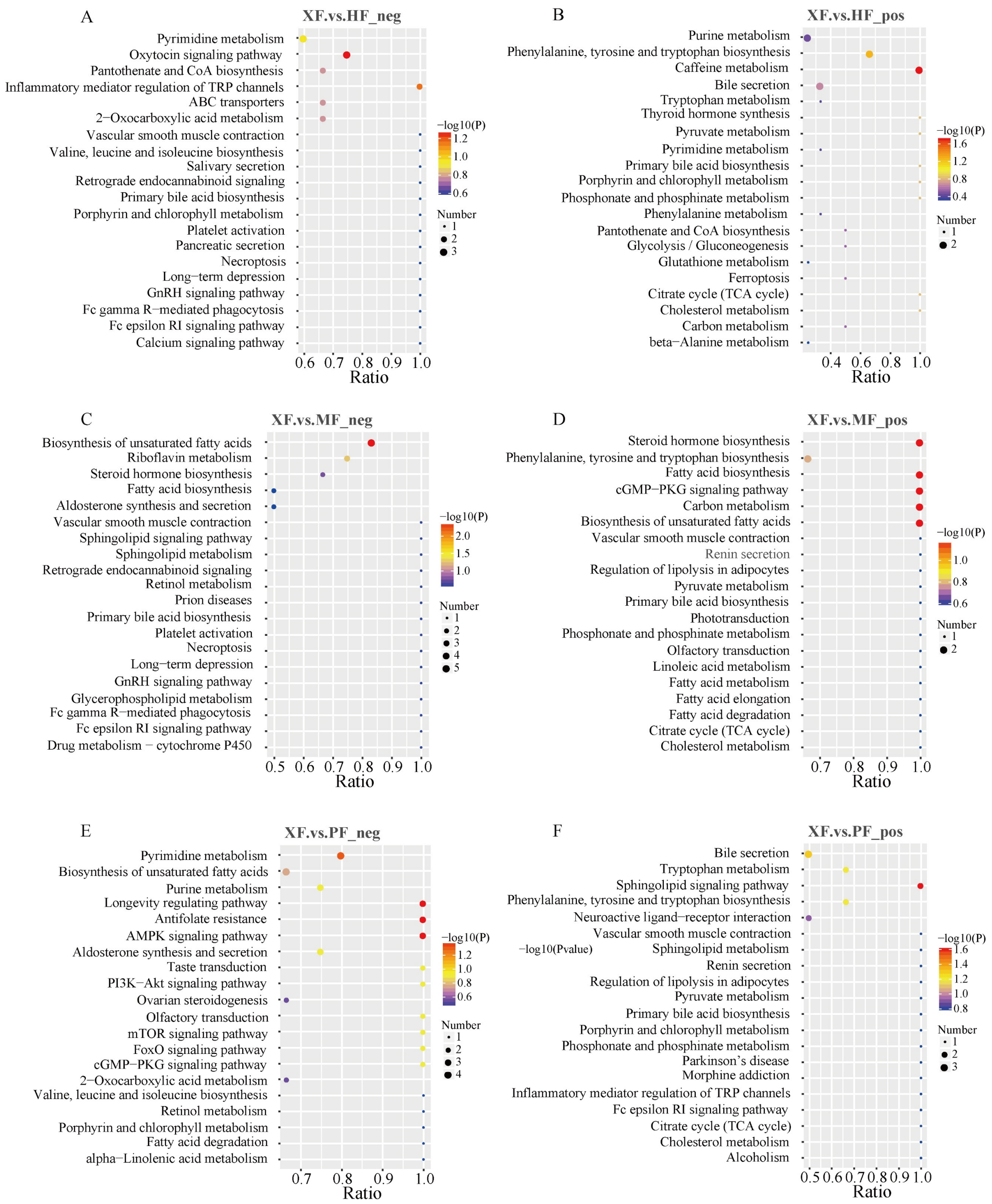

Figure S3. The KEGG pathways of differential metabolites between the four breed groups. (A) XF vs HF_neg; (B) XF vs HF_pos; (C) XF vs MF_neg; (D) XF vs MF_pos; (E) XF vs PF_neg; (F) XF vs PF_pos.
